# Supplementary material for: Patent ductus arteriosus (also non-hemodynamically significant) correlates with poor outcomes in very low birth weight infants. A multicenter cohort study
Source: PLoS One. 2024 Jul 9;19(7):e0306769. doi: 10.1371/journal.pone.0306769 (PMC11233010; doi:10.1371/journal.pone.0306769)
Supplement: Attachment — Submitted filename: PLOS One PDA review.docx [file pone.0306769.s002.docx]

Thank you for the opportunity to review this work. I believe the authors efforts merit publication; however, I have added some areas of clarification which I think would strengthen the present manuscript.

**Methods:**

Chesi and colleagues performed a ~10year prospective, multicenter observational study with the objective of standardizing PDA diagnosis and report associated outcomes for (218) VLBW infants.

Of these, 40% were treated for PDA in the first postnatal week didn’t have HsPDA but exhibited “clinical worsening.”

After adjustment for gestational age, finding include:

Risk of death 15-fold higher among these infants (no US evidence of HsPDA, only clinical suspicion).

Risk of death similar between infants with HsPDA and those without PDA.

I assume that adjustment for GA was an attempt to control for problems of prematurity to address associations between PDA treatment (contributing to or diminishing these outcomes: death, ROP, IVH, BPD etc.). Given that most (>80%) of infants who undergo definitive PDA closure require mechanical ventilation (invasive or non-invasive) I would like to see more data (aside from FiO_2_) in regard to level and duration of respiratory support as these data are competing risks/influenced the observed differences for mortality (15-fold), BPD (6-fold higher) and ROP (18-fold) among infants with HsPDA, without an observed benefit of early closure.

Please include n (%) for invasive/noninvasive respiratory support and high-frequency ventilation utilization to the clinical finding/demographics table. Further, please provide duration of mechanical ventilation for comparison between groups (PDA closed vs. those without PDA).

Discussion line 283ish: Please add to discussion that while PDA diameter has historically been incorporated into these scores, it is a poor indicator of adverse outcomes in isolation. <https://www.sciencedirect.com/science/article/abs/pii/S0378378223001287?via%3Dihub>

290: Indeed, the large challenge for treatment of PDA is agreement on what constitutes a hemodynamically significant PDA and is it pathologic as noted for group E4-flow reversal in descending Ao. Conversely, the watch and wait E2 may be subject to protracted mechanical ventilation and related morbidity/mortality. As such, continued work is needed to elucidate when the “watch” ends and what it the optimal method of definitive closure (TCPC or ligation) based on infant criteria (weight, PDA size, morphology, length, presence of absence of infection etc.)

293: Authors appropriately acknowledge the statistical associations were likely impacted by small sample size and low number of deaths (events).

Please include within your discussion that percutaneous closure has demonstrated efficacy for closing HsPDA with several potential benefits over surgical ligation (mainly avoidance of incision and related problems related to manipulating the lung to access PDA or laryngeal recurrent nerve damage). Moreover, it’s worth noting that the PIVOTAL study may help answer some of these questions. https://www.pivotalstudy.org

Conclusion: I agree with the authors that there are knowledge gaps insofar as criteria, timing, and method of assessing for a HsPDA (including concensus definition) and type of definitive closure that minimize adverse outcomes in this population.
