# Supplementary material for: Patent ductus arteriosus (also non-hemodynamically significant) correlates with poor outcomes in very low birth weight infants. A multicenter cohort study
Source: PLoS One. 2024 Jul 9;19(7):e0306769. doi: 10.1371/journal.pone.0306769 (PMC11233010; doi:10.1371/journal.pone.0306769)
Supplement: Attachment — Submitted filename: Response to reviewers.docx [file pone.0306769.s003.docx]

We warmly thank the editors and referees for their valuable suggestions, and certainly, our manuscript has improved after changes. Please find here a point-by-point reply.

**Journal Requirements:**

1. When submitting your revision, we need you to address these additional requirements.

Please ensure that your manuscript meets PLOS ONE's style requirements, including those for file naming. The PLOS ONE style templates can be found at 
https://journals.plos.org/plosone/s/file?id=wjVg/PLOSOne_formatting_sample_main_body.pdf and 
<https://journals.plos.org/plosone/s/file?id=ba62/PLOSOne_formatting_sample_title_authors_affiliations.pdf>

Changes made.

2. Did you know that depositing data in a repository is associated with up to a 25% citation advantage (https://doi.org/10.1371/journal.pone.0230416)? If you’ve not already done so, consider depositing your raw data in a repository to ensure your work is read, appreciated and cited by the largest possible audience. You’ll also earn an Accessible Data icon on your published paper if you deposit your data in any participating repository ([https://plos.org/open-science/open-data/#accessible-data](https://plos.org/open-science/open-data/#accessible-data)).

We are committed to facilitating responsible and ethical data sharing while ensuring the privacy and confidentiality of sensitive information. We added a data availability statement: the data underlying this research are not publicly available due to the presence of potentially identifying or sensitive patient information. Researchers interested in accessing the data for legitimate research purposes may submit data requests to the corresponding author.

3. In your Data Availability statement, you have not specified where the minimal data set underlying the results described in your manuscript can be found. PLOS defines a study's minimal data set as the underlying data used to reach the conclusions drawn in the manuscript and any additional data required to replicate the reported study findings in their entirety. All PLOS journals require that the minimal data set be made fully available. For more information about our data policy, please see http://journals.plos.org/plosone/s/data-availability.

Upon re-submitting your revised manuscript, please upload your study’s minimal underlying data set as either Supporting Information files or to a stable, public repository and include the relevant URLs, DOIs, or accession numbers within your revised cover letter. For a list of acceptable repositories, please see http://journals.plos.org/plosone/s/data-availability#loc-recommended-repositories. Any potentially identifying patient information must be fully anonymized.

Important: If there are ethical or legal restrictions to sharing your data publicly, please explain these restrictions in detail. Please see our guidelines for more information on what we consider unacceptable restrictions to publicly sharing data: http://journals.plos.org/plosone/s/data-availability#loc-unacceptable-data-access-restrictions. Note that it is not acceptable for the authors to be the sole named individuals responsible for ensuring data access.We will update your Data Availability statement to reflect the information you provide in your cover letter.

We explained reasons (including privacy and ethical considerations) in the data availability statement.

**Reviewers' comments:**

Reviewer's Responses to Questions

**Comments to the Author**

1. Is the manuscript technically sound, and do the data support the conclusions?

The manuscript must describe a technically sound piece of scientific research with data that supports the conclusions. Experiments must have been conducted rigorously, with appropriate controls, replication, and sample sizes. The conclusions must be drawn appropriately based on the data presented.

Reviewer #1: Yes

Reviewer #2: Yes

2. Has the statistical analysis been performed appropriately and rigorously?

Reviewer #1: Yes

Reviewer #2: I Don't Know

3. Have the authors made all data underlying the findings in their manuscript fully available?

The [PLOS Data policy](http://www.plosone.org/static/policies.action#sharing) requires authors to make all data underlying the findings described in their manuscript fully available without restriction, with rare exception (please refer to the Data Availability Statement in the manuscript PDF file). The data should be provided as part of the manuscript or its supporting information, or deposited to a public repository. For example, in addition to summary statistics, the data points behind means, medians and variance measures should be available. If there are restrictions on publicly sharing data—e.g. participant privacy or use of data from a third party—those must be specified.

Reviewer #1: Yes

Reviewer #2: Yes

4. Is the manuscript presented in an intelligible fashion and written in standard English?

PLOS ONE does not copyedit accepted manuscripts, so the language in submitted articles must be clear, correct, and unambiguous. Any typographical or grammatical errors should be corrected at revision, so please note any specific errors here.

Reviewer #1: No

Reviewer #2: Yes

5. Review Comments to the Author

Please use the space provided to explain your answers to the questions above. You may also include additional comments for the author, including concerns about dual publication, research ethics, or publication ethics. (Please upload your review as an attachment if it exceeds 20,000 characters)

Reviewer #1: Dear Authors,

I evaluated the study named “Patent ductus arteriosus: diagnosis, management and adverse neonatal outcomes in preterm Very Low Birth Weight Infants. Area-based prospective multicenter observational study”, with great pleasure and interest. This is a relevant study. However, the manuscript may be suitable for publication after the correction is made. I listed my recommendations and comments below

Overall assessment

In this study, the authors found that when clinical and echocardiographic collaboration were used in the treatment of PDA, about 40 % of patients were treated as medical due to deterioration of the clinic in asymptomatic patients with PDA. Prevalence of death, intraventricular hemorrhage, retinopathy of prematurity, and bronchopulmonary dysplasia were higher in patients with PDA

Patent ductus arteriosus is one of the most common neonatal morbidity in neonatal intensive care but the relationship between the ductus arteriosus and neonatal morbidity remains unclear despite many research papers. Echocardiography is the gold standard for identifying significant PDA. However, every hs-PDA with echocardiographic findings may not cause significant clinical effects. Besides, there is still ongoing debate about the treatment approaches such as conservative? medical treatment? observation?. Therefore, the approaches vary among neonatal intensive care units. In this context, The study is much more important in terms of emphasizing the significance of the individualized approach in the current treatment of PDA


1. The title is not exactly reflective of the study or its content. could Authors please write a more appropriate and clear title to attract readers and encompass your study

Thanks for your suggestion: we changed the title to be more exhaustive and informative.

The abstract conclusion should involve summarising the key points of the study and presenting the implications of its findings. The conclusion could be revised to get a clear understanding and should be far more specific to the study.

Changed as requested

Introduction
The hypothesis of the article is not clear. Authors should determine a more concise, testable, and specific statement of the research question. The hypothesis should directly support the main research question. This should establish a clear link between the hypoyhesis and the purpose of the study

Thanks for the suggestion. We changed the structure of the introduction.

Methodology
Line 113 (An observational prospective area-based study…) may be more appropriate if changed as a multicenter observational study.

Changed, thanks.

Authors don't have a control group, why did the authors consider having a control group that was evaluated with echocardiography performed by a cardiologist?

In our NICUs, cardiologists don’t perform echocardiography on newborns. For cases of complex heart diseases, we refer to the Level III Pediatric Cardiology Center in Bologna.

Line 114: authors should not write the aim of the study in the method section.

Changed, thanks

Line 118 For data collection purposes, standardized form… what was the component standardized form used by the authors? The authors should elaborate on its content, providing more details and explanations

Added.

Ultrasound and clinical grading of PDA
The authors should provide some insight into the reliability of the neonatologist who conducted the functional echocardiography. For example, how or how many times was PDA measured (one or more), which can be influenced by straining, crying, and cold environment, Which measurement was confirmed PDA? single or the average of measurements?

Added

Please authors should give definitions of AKI, hypotension, and other morbidities such as NEC, BPD, and ROP in the method section. Authors should add these definitions and references to the methods

We included the definitions of unfavorable neonatal outcomes and relevant citations in the methods section. The definitions of AKI and hypotension are in the table legend where they appear (in the methods section), for greater reading convenience.

Short-term clinical outcomes
Authors should state other morbidities related to PDA such as respiratory support (CPAP and mechanical ventilation), surfactant administration, surfactant doses, extubation failure, postnatal steroids…

We did not include variables such as surfactant administration among PDA outcomes because, according to the literature, it is less associated with PDA and more with the severity of respiratory distress syndrome. As for postnatal steroids, these appeared to be a result of the development of bronchopulmonary dysplasia (BPD).

Line 147: Data analysis Statistical analysis was carried out using SPSS software 23.0. … which test was used to assess the normality distribution of data. The appropriate statistical method should be written for each specific situation

We did not test the distribution of data because for inference purposes we relied on nonparametric tests and logistic regression models, which do not require normal distributed data to be effective. Regarding the estimated regression models, the normal distribution of the residuals was checked by visually assessing the residuals Q-Q plot. We added more details about this in the Data Analysis paragraph.

Line 147: Continuous variables were expressed as the means ± standard deviations or medians and interquartile ranges. Authors presented all continuous data as median and interquartile so you don’t need to use means and standard deviation

Ok, correct.

Figure 1 is very interesting. Authors should add Figure 1 to the methods section, explaining how to make or comment because it will guide readers and enhance the overall understanding of the figure’s significance in the context of the research

Ok. Please find the figure 1 cited in the methods section (paragraph: US and clinical grading of PDA)

Line 152: “Confidence Interval (CI) was 0.95; when p < 0.05, tests were considered
statistically significant” should be the final sentence for the data analysis section.

Ok

Line 154: ‘The Strengthening the Reporting of Observational Studies in Epidemiology (STROBE) reporting guidelines were followed for this study’ The sentence seems unnecessary because authors must follow it in observational studies to structure their manuscript, making it easier to be understood

Ok, we removed the sentence.

Line 178: Demographic and further maternal and neonatal characteristics according to gestational age (GA < 26 versus . 26 wks gestation) are shown in Table 3. This sentence might have been changed to “Demographic, maternal and neonatal characteristics are shown in Table 3.

Ok


The English language must be revised throughout the text. If the manuscript is checked by a Language Editing in terms of grammar and punctuation, it would be better

We have refined the English language throughout the entire manuscript, aiming for improved clarity and accuracy. We are at your disposal for any additional revisions that may be necessary

In Table 3, there is no need to give as a column in table 3 because the missing data is exclusion criteria

We reported these data because as per the STROBE guidelines number of participants with missing data for each variable of interest should be indicated

Line 236 ‘The Fisher exact test was used to calculate the p-value for each variable except the Apgar score for which the Kruskal-Wallis¥ test is applied’ should be placed in the statistical section
this sentence need not be repeated under Table 3

Ok, changed

Line 242 Among 207 surviving infants at 36 weeks’ gestation. Eleven patients were excluded because they…. Please Please put a comma instead of a dot before eleven patients.

Ok, changed

There is the same error in Line 245, which must be corrected.

Ok

Line 343 Figure 2 Flow diagram of the study: ultrasound studies and findings.
there is no need to write “Ultrasound studies and findings “

Ok

Figure 4:
Flow diagram of patients treated for PDA. Early and/or late medical and/or surgical
treatment are reported. of PDA. PDA: patent ductus arteriosus. Early PDA treatment: any treatment in the first week of life. Late PDA treatment: any treatment after the first week of life.
Please revise to get a clear understanding. You may change as “Flow diagram of patients treated with medical or surgical ligation”

Ok

Discussion
The authors should begin the discussion by summarising the main findings of their study concisely. For example, our study shows….
Authors should make much more comprehensive explanations about the clinical implications of their findings. How might these results impact the monitoring and treatment guidelines of PDA?
Please ensure that clear messages will be accessible to broad readers related to your study

Thanks for the suggestion: we modified the discussion, highlighting clinical implications.


Conclusion

Please focus on summarising key findings without unnecessary details

We simplified the conclusions, reducing some numerical data.

Reviewer #2: Thank you for the opportunity to review this work. I believe the authors efforts merit publication; however, I have added some areas of clarification which I think would strengthen the present manuscript.

Methods:
Chesi and colleagues performed a ~10year prospective, multicenter observational study with the objective of standardizing PDA diagnosis and report associated outcomes for (218) VLBW infants.

Of these, 40% were treated for PDA in the first postnatal week didn’t have HsPDA but exhibited “clinical worsening.”

After adjustment for gestational age, finding include:

Risk of death 15-fold higher among these infants (no US evidence of HsPDA, only clinical suspicion).

Risk of death similar between infants with HsPDA and those without PDA.

I assume that adjustment for GA was an attempt to control for problems of prematurity to address associations between PDA treatment (contributing to or diminishing these outcomes: death, ROP, IVH, BPD etc.). Given that most (>80%) of infants who undergo definitive PDA closure require mechanical ventilation (invasive or non-invasive) I would like to see more data (aside from FiO2) in regard to level and duration of respiratory support as these data are competing risks/influenced the observed differences for mortality (15-fold), BPD (6-fold higher) and ROP (18-fold) among infants with HsPDA, without an observed benefit of early closure.

Please include n (%) for invasive/noninvasive respiratory support and high-frequency ventilation utilization to the clinical finding/demographics table. Further, please provide duration of mechanical ventilation for comparison between groups (PDA closed vs. those without PDA).

We are sorry, but this is a prospective study and unfortunately these data were not present in the data collection form.

Discussion line 283ish: Please add to discussion that while PDA diameter has historically been incorporated into these scores, it is a poor indicator of adverse outcomes in isolation. <https://www.sciencedirect.com/science/article/abs/pii/S0378378223001287?via%3Dihub>

Thanks, we have integrated the suggested point with relevant citation.


290: Indeed, the large challenge for treatment of PDA is agreement on what constitutes a hemodynamically significant PDA and is it pathologic as noted for group E4-flow reversal in descending Ao. Conversely, the watch and wait E2 may be subject to protracted mechanical ventilation and related morbidity/mortality. As such, continued work is needed to elucidate when the “watch” ends and what it the optimal method of definitive closure (TCPC or ligation) based on infant criteria (weight, PDA size, morphology, length, presence of absence of infection etc.)

Thanks for your suggestion: we included these considerations in both abstract and discussion.

293: Authors appropriately acknowledge the statistical associations were likely impacted by small sample size and low number of deaths (events).

Thanks

Please include within your discussion that percutaneous closure has demonstrated efficacy for closing HsPDA with several potential benefits over surgical ligation (mainly avoidance of incision and related problems related to manipulating the lung to access PDA or laryngeal recurrent nerve damage). Moreover, it’s worth noting that the PIVOTAL study may help answer some of these questions. <https://www.pivotalstudy.org>

Ok, added.


Conclusion: I agree with the authors that there are knowledge gaps insofar as criteria, timing, and method of assessing for a HsPDA (including concensus definition) and type of definitive closure that minimize adverse outcomes in this population.

6. PLOS authors have the option to publish the peer review history of their article ([what does this mean?](https://journals.plos.org/plosone/s/editorial-and-peer-review-process#loc-peer-review-history)). If published, this will include your full peer review and any attached files.


If you choose “no”, your identity will remain anonymous but your review may still be made public.


**Do you want your identity to be public for this peer review?** For information about this choice, including consent withdrawal, please see our [Privacy Policy](https://www.plos.org/privacy-policy).

Reviewer #1: **Yes:** Associate Professor Fatih BOLAT, MD

Reviewer #2: No
