# Supplementary material for: Patent ductus arteriosus (also non-hemodynamically significant) correlates with poor outcomes in very low birth weight infants. A multicenter cohort study
Source: PLoS One. 2024 Jul 9;19(7):e0306769. doi: 10.1371/journal.pone.0306769 (PMC11233010; doi:10.1371/journal.pone.0306769)
Supplement: Attachment — Submitted filename: Response to Reviewer.docx [file pone.0306769.s004.docx]

We warmly thank the referee for his valuable suggestions, and certainly, our manuscript has improved after changes. Please find here a point-by-point reply.

The title still does not exactly reflect the study content. Please write a more appropriate and clear title that is associated with your study

We changed it. Hope this is clearer.

Abstract
Line 52: …Patent Ductus Arteriosus (patent ductus arteriosus (PDA) should be written as lowercase letter

Correct, changed as suggested.

Line 53:… in preterm Very Low Birth Weight Infants (VLBW, birth weight < 1500 g)
Int
it would be more appropriate if changed as `? in very low birth weight infants’

Changed as suggested.

Line 57:The association between ultrasound grading of PDA and adverse neonatal outcomes was evaluated after correction for gestational age (GA)…. you could delete GA because you only use it once in the abstract

Changed as suggested.

Line 62:…. in the first postnatal week, up to 40% did not have hsPDA at ultrasound (US), but… US" should not be abbreviated as it was not mentioned previously.

Ok, we eliminated the abbreviation in the abstract.

Line 72: ‘The occurrence of BPD was 6-fold higher among neonates with hsPDA, with no apparent beneficial role of early PDA treatment. The risk of IVH (grade ≥ 3) and ROP (grade ≥ 3) were 8-fold a…..’ Please consider providing exact percentages or numerical values when referring to increased risk
Here, we refrained from providing an exact percentage for the risk of BPD in neonates with hsPDA due to the inclusion of two categories with different odds ratios (6.75 and 6.46 in E3 and E4 infants, respectively). While precise numerical values are reported in the results section, presenting two numerical values in the abstract may hinder immediate comprehension for the reader. Conversely, we have included the precise numerical data for IVH grade ≥3. For ROP ≥3, we have not altered the number because 17.97 may accurately approximate to 18.

Line 67: In the result section of the abstract, there should be no comment. The authors may add the comments to the conclusion section of the abstract. The conclusion of the abstract should be written as a subheading apart from the result section.

Correct, we have changed as suggested.

Introduction
There are some typographical and grammatical errors like these:
Line 102: Many investigators analyzed the hemodynamic significance of PDA, either by evaluating only echocardiographic indexes [14-16], or by using both clinical and US composite scores [17-19].
Line 104: “The term “hemodynamically significant patent ductus arteriosus 104 (hsPDA)” has being been increasingly used:..”plesase delete "being"

Ok, thanks.

The language is generally clear, but some sentences could be more concise. Please consider a thorough proofread for grammatical accuracy and clarity.

Ok, we have checked the manuscript and made changes for better clarity.


METHODS
Line 183:…(loading and maintenance dose 10 and 5 mg/kg/die….maintenance dose 0.2 and 0.1 mg/kg/die…. These sentences need to be changed to a clearer definition, die converting to day (to be more understandable for the reader)

Done

RESULT
Complete antenatal steroides (correct it as ‘antenatal steroids’) and write

Done

Table 3:
Those percentage values need to be corrected: For example, 19/42 is 45.2% but it is written as 56% in Table 3. Cesarean section, Vaginal delivery, Chorioamnionitis, Maternal hypertension/eclampsia, Gestational diabetes…The numbers seem to have been calculated incorrectly.Mistakes should be corrected throughout the text, tables, and figures.

Percentages are calculated excluding missing cases. We have double checked.

You should standardize all the numbers throughout the paper and either round them down or round them up to one decimal place (some are rounded up, some are in decimals, some are whole numbers)
Line 228: Overall, 70 of 218 neonates (32.1%).. please standardize the numbers:. … not 32.1% but 32%. All of them need to be in the same numerical type.

Ok, done.

Lin 237: no signs of 237 pulmonary over circulation nor systemic hypoperfusion—could you change it as ‘no sign of pulmonary over circulation or systemic hypoperfusion’ or use ‘neither pulmonary over circulation nor systemic hypoperfusion’

Done

Line 245: The total number of patients treated is not 61 patients (when I sum up, it is 60 patients)
Please check all of the numbers and percentages thoroughly again

Early treated patients were 61, comprising 60 receiving medical treatment and 1 undergoing surgical ligation (see also Figure 4 and line 231).

Again, the numbers in Figure 2 should be standardized as previously described.

Changed as requested.

Line 261:’ …with median duration of medical treatment lasting 3.5 days.’ How did the median duration of medical treatment last 3.5 days? Didn't you give a 3-day cure treatment?

The duration of medical treatment may vary (i.e. ibuprofen versus acetaminophen). Median duration of early medical treatment was 3 days, while median duration of late medical treatment was 3.5 days.

Line 263: please write the number without decimals.

Corrected.

Discussion
Line 308: In the study, the authors found that post-ligation syndrome was twice as high
How do you explain this situation?

This discrepancy may be because we defined post-ligation syndrome not as the need for vasopressors within 72 hours post-procedure, but as a reduction in cardiac output on ultrasound within 6 hours of the procedure, possibly including mild cases. No patient died from post-ligation syndrome. We added this piece of information in the text.
