# Supplementary material for: Patent ductus arteriosus (also non-hemodynamically significant) correlates with poor outcomes in very low birth weight infants. A multicenter cohort study
Source: PLoS One. 2024 Jul 9;19(7):e0306769. doi: 10.1371/journal.pone.0306769 (PMC11233010; doi:10.1371/journal.pone.0306769)
Supplement: Attachment — Submitted filename: Response to Reviewer.docx [file pone.0306769.s005.docx]

We extend our sincere gratitude to the reviewer and the editors for dedicating their time and for carefully considering our manuscript. Presented below is a detailed point-by-point response.

1. Please condense the conclusion in the abstract, as it is currently too long to provide a concise conclusion

*Ok, done.*

2. Line 55: please change as “Multicenter prospective observational study was conducted in Emilia Romagna from”

*Ok. Done*

3. Line 58:.” A diagnosis of hemodynamically significant (hsPDA) was established when the PDA diameter was ≥ 1.6 mm at the pulmonary end with growing or pulsatile flow, and at least 2 of 3 indexes of pulmonary overcirculation and/or systemic hypoperfusion were present” should be added to study design

*Corrected as suggested*

3) LİNE 83: PDA can cause result in pulmonary overcirculation and systemic… Please correct

Thanks, corrected

4. Line 103... PDA by evaluating either only…grammar error

*Ok. We made the sentence simpler.*

4) please calculate the ratio accurately, especially in Table 3

The suggested corrections have not been made in Table 3 “Those percentage values need to be corrected: For example, 19/42 is 45.2% but it is written as 56% in Table 3. Cesarean section, Vaginal delivery, Chorioamnionitis, Maternal hypertension/eclampsia, Gestational diabetes…The numbers seem to have been calculated incorrectly.

*Here we employed the "Complete-case analysis" method to deal with missing data. When data was missing, the case was excluded from the analysis to avoid bias. For example, as indicated by the reviewer, in the case of antenatal steroids, data was missing in 8 out of 42 patients < 26 weeks: thus, we believe the correct percentage remains 19/34 (=56%), not 19/42 (45%), because 8 cases are missing and thus cannot be included in the analysis (referred to as complete-case analysis). Therefore, our choice was deliberate, not indicative of numerical errors. We have modified the explication in the footnotes for better clarity. However, we are open to a reassessment of our decision if you deem it appropriate.*

4) The manuscript has grammatical errors that should be corrected. For example. Line 55

…. Multicenter prospective observational study was conducted in Emilia Romagna from 56 March 2018 to October 2019.

Please comprehensively check by a native speaker

*This was a nominal phrase, to save words. However, we had a second extended English editing from a native speaker.*

We thank the reviewer and the editors, as thanks to their time and effort, the quality of our manuscript has certainly increased.
